# Capsaicin: beyond TRPV1

**DOI:** 10.3389/fnut.2025.1594742

**Published:** 2025-05-29

**Authors:** Rebeca Juárez-Contreras, Edgardo Mota-Carrillo, Angelica Piedra-Ramírez, Daniel Farías-Sánchez, Ricardo González-Ramírez, Sara Luz Morales-Lázaro

**Affiliations:** ^1^División de Neurociencias, Instituto de Fisiología Celular, Universidad Nacional Autónoma de México, Mexico City, Mexico; ^2^Programa de Posgrado en Ciencias Biológicas, Unidad de Posgrado, Universidad Nacional Autónoma de México, Mexico City, Mexico; ^3^Programa de Doctorado en Ciencias Biomédicas, Unidad de Posgrado, Universidad Nacional Autónoma de México, Mexico City, Mexico; ^4^Programa de Maestría y Doctorado en Ciencias Bioquímicas, Unidad de Posgrado, Universidad Nacional Autónoma de México, Mexico City, Mexico; ^5^Departamento de Biología Molecular e Histocompatibilidad, Hospital General "Dr. Manuel Gea González", Mexico City, Mexico; ^6^Centro de Investigación Sobre el Envejecimiento, CINVESTAV, Mexico City, Mexico

**Keywords:** capsaicin, amphiphilic molecule, membrane properties, anti-cancer, anti-inflammatory, TRPV1-independent

## Abstract

Capsaicin, the chili-pungent compound, has a peculiar chemical structure that allows it to impact mammalian physiology. Besides its classical effects through activating the Transient Receptor Potential Vanilloid type 1 (TRPV1), growing experimental evidence demonstrates that capsaicin has pleiotropic actions in a TRPV1-independent manner. These effects are achieved by modifying the membrane features or interacting with unclassical putative molecular targets. Here, we will summarize the representative information related to the capsaicin actions through unclassical and TRPV1-independent molecular mechanisms, and we will discuss the impact of these effects on non-neuronal cells and mammalian physiology.

## 1 Capsaicin and TRPV: an overview

Capsaicin (trans-8 methyl-N-vanillyl-6-nonenamide, C_18_H_27_NO_3_) is the main pungent component of plants of the genus *Capsicum*. It is a crystalline compound with a molecular weight of 305.42 g/mol, a melting point of 65°C, freely soluble in alcohols (partition coefficient K_ow_ ~ 3) and has intrinsic fluorescence in aqueous media (1–3).

**Figure 1:**
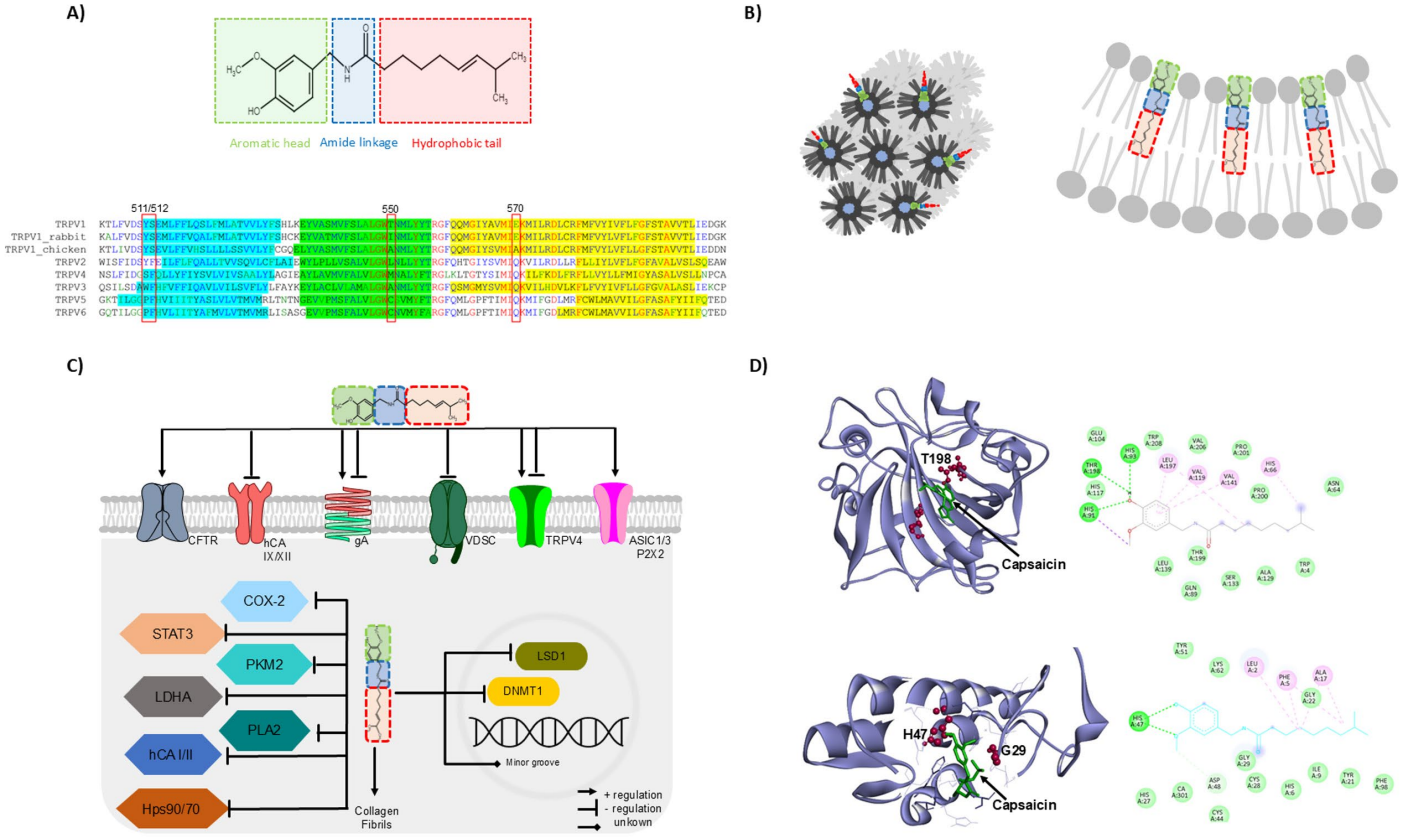
Capsaicin unclassical molecular targets. **(A)** Capsaicin structural features: flexible alkyl tail- (red), neck- (blue), and a head-domain (green) form the amide and vanillyl moieties (upper). Sequence alignment of the human TRPV1 “vanilloid pocket” with chicken and rabbit TRPV1 and human TRPV2-V6; capsaicin binds to Y511, S512, T550 and E570 from human TRPV1, the amino acids highlighted in aqua, green and yellow represents the transmembrane segments S3, S4 and S5, respectively (lower) **(B)** Capsaicin insertion into the membrane produces the organization of phospholipids in the hexagonal HII phase or negative curvature of the membrane. **(C)** Schematic representation of capsaicin effects toward unclassical molecular targets. **(D)** Molecular docking analysis of capsaicin with two unclassical partners. Capsaicin interaction with Human carbonic anhydrase XII (hCA XII, PDB: 5MSA), molecular simulation shows H-bond formed between capsaicin and T198 of hCA XII (upper). PLA2-capsaicin complexes throughout the MD simulation showing the direct interaction of some residues of PLA2 (PDB:1KQU) with capsaicin (dotted lines, lower). The molecular dockings were performed using the Autodock-Vina software (65–67) and the image was prepared in Discovery Studio Visualizer (v25.1.0.24284, BIOVIA, Dassault Systèmes, 2025).

Structurally, capsaicin is an amphiphilic molecule containing a flexible alkyl tail forming Van der Waals interactions with hydrophobic environments and two rigid polar regions resembling a neck-and a head-domain formed by the amide and vanillyl moieties, respectively, these polar domains establish hydrogen bonds with other molecules (4), Figure 1A (upper). The structural features of capsaicin allow high-affinity interaction with its classical target (5), the Transient Receptor Potential Vanilloid 1 (TRPV1), resulting in the pungency produced when eating chili. The TRPV1 is abundantly expressed in the nociceptive sensory neurons of the Dorsal Root and Trigeminal Ganglia (DRGs and TGs, respectively), playing a crucial role in transducing environmental signals as temperature (≥ 42°C) and chemical compounds (as capsaicin) (6).

TRPV1 belongs to the Transient Receptor Potential Vanilloid (TRPV) subfamily, which is integrated by six members (7). This subfamily was named as its first identified member, TRPV1, the capsaicin receptor (6). Subsequently, the discovery of other receptors with high homology in their sequences were grouped as the vanilloid subfamily of TRP channels. The mechanism of action of TRPV1-TRPV4 channels is to allow the passage of cations such as Na^+^ and Ca^2+^, whereas TRPV5 and TRPV6 are highly selective for Ca^2+^, interestingly, some of them are crucial mediators of the nociception, sensing chemical and thermal stimuli, for example, TRPV3 and TRPV4 are activated by warm temperature. In contrast, noxious temperature activates TRPV1 and TRPV2 channels. TRPV5 and TRPV6 are pivotal in the physiology of kidneys and airways, specifically regulating the Ca^2+^ homeostasis in these tissues.

These receptors are tetrameric non-selective cation channels essential as molecular transducers of physical and chemical signals. Each monomer is a six-pass transmembrane (S1-S6) protein with intracellular N-and C-ends and an ion pore former loop located between S5-S6; the N-end contains six ankyrin repeats, a structural feature shared only by members of this subfamily, while the C-end contains a signature sequence named the TRP box (7).

The 3D structure of all TRPV channels has been resolved through cryo-electron microscopy, a turning point for understanding the structure and its relationship with their function (8). These studies structurally confirmed their tetrameric assembly with the central ion pathway between S5-S6 flanked by the voltage-sensor-like domain (S1-S4) (8). A complete understanding of the relationship between their structure and function has been evidenced through electrophysiological studies combined with site-directed mutagenesis and detailing the fine regulation of the gating of these channels and the structural requirements of their ligands impacting their function (8).

The overall activation mechanism of these ion channels located at the plasma membrane requires a specific stimulus producing a structural conformational change impacting in their lower gate and allowing the ion flux through them (8). Interestingly, capsaicin produces this effect through the selective activation of the TRPV1; this specific effect is produced through capsaicin’s direct interaction with specific amino acids contained in a site called the “vanilloid pocket,” which is located in the S3-S4 and S4-S5 linker of TRPV1 (5).

The interaction between capsaicin and TRPV1 requires the former to acquire a “tail up and head down” configuration to insert between the S3-S4 of the receptor, establishing Van der Walls interactions with some transmembrane residues. Then, the formation of H-bound between the amide group of capsaicin and threonine 550 (T550) of TRPV1 stabilizes their binding. Also, H-bonds are formed between the vanillyl group and amino acids located in the lipid-water interface (Y511, S512), strengthening the molecular interaction. Finally, the capsaicin head binds to E570 of the receptor, facilitating the gating of the channel (5).

The TRPV2-V6 lack of the “vanilloid pocket,” thus these channels are not directly activated by capsaicin. Furthermore, differences in this vanilloid pocket have also been found on TRPV1 of different species, allowing rabbit and chicken to be less sensitive or insensitive to capsaicin pungency, respectively, (9–11), Figure 1A (lower).

The knowledge of capsaicin binding to its classical receptor, the TRPV1, highlighted that specific structural requirements are needed in the proteins to be regulated by the unclassical capsaicin effects. Interestingly, several reports show that capsaicin exerts its effects by interacting with other molecular targets. Here, we review the unclassical and TRPV1-independent actions of capsaicin, highlighting its impact on non-neuronal cells and implications for mammalian physiology.

## 2 Capsaicin: an amphiphilic compound modifying the membrane properties and ion channel’s function

Capsaicin inserts into lipid bilayers adopting a head-to-head group (vanillyl moiety towards phospholipid head) and tail-to-tail (alkyl and acyl chains aligned) configurations, modifying the stiffness and fluidity of the membrane at high micromolar concentrations and promoting negative curvature and the organization of phospholipids in the hexagonal HII phase (12–14), Figure 1B.

The NMR spectroscopic analysis performed on synthetic bilayers composed of 1,2-palmitoyl-sn-glycero-3-phosphocholine (DPPC) showed that capsaicin locates at the upper of the monolayer with the polar head close to the lipid-water interface and its hydrophobic tail next to the fatty acyl chain of phospholipid, producing fluidification and disorganization of the membrane (15). Similarly, the capsaicin insertion into neuro-mimetic membranes (composed of 1-palmitoyl-2-oleoyl phosphatidylcholine, sphingomyelin and cholesterol) modifies the membrane fluidity in a concentration-dependent manner, increasing and decreasing the fluidity at 50–200 μM and 250 μM of capsaicin, respectively (12, 16).

Capsaicin membrane insertion modulates the function of some transmembrane proteins via membrane perturbations rather than specific protein binding (17, 18). X-ray diffraction experiments demonstrated that capsaicin inserted on synthetic membranes made of 1,2-dimysristoyl-sn-glycero-3-phosphocholine increases the density of head groups, thinning and bending the membranes (18). These membrane perturbations affect the function of some ion channels; for example, experiments performed on *Xenopus* oocytes expressing the ASIC1a or ASIC3 channels (which are activated by pH 6.5) or the P2X2 receptor (activated by ATP) demonstrated that capsaicin produced an increase in their peak current amplitude evocated by their specific ligands (18).

Capsaicin also affects the functional organization of the bacterial gramicidin A channel (gA) inserted into the synthetic lipid bilayer; these channels are trans-bilayer dimers formed by β6.3-helical subunits from each leaflet, the formation of functional gA dimers are producing at 10–100 μM of capsaicin, since capsaicin increases the membrane fluidity allowing the dimer formation (13). However, higher capsaicin concentrations (250 μM) decreased the gA inward currents of the channels expressed in *Xenopus* oocytes (18). These data indicate that capsaicin actions over membrane properties are concentration-dependent, influencing positively or negatively ion channel function, Figure 1C.

### 2.1 Capsaicin membrane insertion affects the cardiac ion channel’s function

Capsaicin effects on ion channel function have been tested at high capsaicin concentrations and in TRPV1-free cell systems, ensuring that capsaicin actions are TRPV1-independent. Under these conditions, it has been also reported that capsaicin modifies the function of some voltage-dependent sodium channels (VDSCs), such as the Nav1.5, a mechanosensitive channel highly expressed in cardiac tissue that drives the excitability of cardiomyocytes (19). High capsaicin concentration (30 to 100 μM) negatively regulates the Nav1.5 channel activation, an effect produced by decreasing the lipid bilayer’s stiffness and disjoining force which stabilizes the inactive state of this channel (13, 17). Remarkably, capsaicin also reduces the Nav1.5 peak current produced by pressure and shear stress without affecting the channel’s voltage-dependence gating, suggesting that the mechanism implied changes on the membrane properties (13, 20, 21).

Capsaicin also inhibits inward (Na^+^ and Ca^2+^) and outward (K^+^) currents from rabbit ventricular myocytes, impacting the cardiac excitability (21). Remarkably, the capsaicin effect over these channels is gradual, reaching steady-state inhibition at 3 to 6 min after capsaicin application, and partially reversed. The co-application of capsaicin and capsazepine (a TRPV1 antagonist) preserved capsaicin’s inhibitory effect on these channels, pointing a TRPV1-independent mechanism. Thus, the Na^+^, K^+^ and Ca^2+^ currents inhibition by capsaicin are through the modification of membrane properties. These results prompted us to question the physiological role of capsaicin in cardiac tissues. Considering that cardiac excitability needs the coordination of depolarization and repolarization events to propagate the electrical activity, the accurate ion channel function is strictly necessary. The computer modeling of rabbit ventricular action potential with the rectifier K^+^ currents inhibited at low capsaicin concentration emulate marked prolongation of action potential duration at 50% of the amplitude (APD50), while the inhibition of voltage-gated Na^+^ and Ca^2+^ and inward rectifier K^+^ currents at high capsaicin concentrations leads to a shortening of APD50 (21); prolongation and shortening of APD50 may cause cardiac arrhythmia (22). Thus, capsaicin insertion into the membrane of cardiomyocytes affects their excitability, having harmful effects.

## 3 The capsaicin unclassical targets

### 3.1 Capsaicin interaction with enzymes: putative anti-cancer effects

In recent years, the number of reports about capsaicin’s role in different kinds of cancer has increased. Interestingly, a recent report demonstrated that capsaicin interacts with the transmembrane isoforms *h*CA IX and *h*CA XII, these are tumor-associated human Carbonic Anhydrases (*h*CAs) (with a K_I_ of 0.28 and 0.064 μM, respectively) (23). These metalloenzymes catalyze the reversible hydration of CO_2_ to bicarbonate and protons, maintaining the suitable acid–base balance in the tissues (24). Capsaicin interacts with the active site of these transmembrane isoforms and encloses its alkyl chain to the hydrophobic surface of the active site. The capsaicin amide group establishes electrostatic interactions with zinc ion and H-bond between its carbonyl group and the backbone of T200 and T198 from *h*CA IX and *h*CA XII, respectively, Figure 1D upper. The biological relevance of the capsaicin inhibitory effect over *h*CA IX and *h*CA XII was probed in the A549 cell line (adenocarcinoma human alveolar basal epithelial cells) which overexpress these enzymes, the capsaicin treatment decreases the high cell migration profile; furthermore, *h*CA IX upregulates the MMP9 expression and capsaicin treatment reverted it (23). Other reports show that capsaicin also inhibits the overactivation of the cytosolic isoenzymes I and II (*h*CA I and *h*CA II, respectively) (25, 26).

The capsaicin anticancer effects have also been related to the inhibition of the tumor-associate NADH oxidase (tNOX), which displays specific and lot of expression in the cell surface of several types of cancer (27). These effects have been reported in malignant melanoma, bladder carcinoma, and some gastric cancer cells, among others (28–30). The treatment of these cells with capsaicin produces cell cycle arrest and apoptosis. Furthermore, direct interaction between capsaicin and tNOX was demonstrated using cellular thermal shift assay (CETSA). This molecular association triggers tNOX to degradation, affecting SIRT1 and consequently producing apoptosis (28–30).

Capsaicin also produces cytotoxic effects in cancer cells through the inhibition of heat shock proteins 90 and 70 (Hsp90 and Hsp70) overexpressed in certain tumors (31). These proteins form a co-chaperone helper system that folds mutant proteins, resulting in a favorable tumor phenotype. Remarkably, capsaicin directly interacts with the ATP-binding pocket located at the N-terminus of Hsp90 (32). This interaction blocks the ATPase activity of Hsp90 producing the Hsp70 uncoupling and its lysosomal degradation. Furthermore, cancer cell lines co-treated with capsaicin and a well-characterized Hsp90 inhibitor, 17-AAG, showed improved anti-tumor effects (32).

The capsaicin role in cancer also extends to the regulation of enzymes implied in the histone methylation. Specifically, capsaicin acts as an inhibitor of the Lysine Specific Demethylase 1A (KDM1A/LSD1) (33), which is overexpressed in different kinds of cancer (34). Capsaicin reversibly and specifically inhibits LSD1 with an IC50 = 0.6 ± 0.04 μM. Docking analysis suggested that capsaicin interacts with the FAD binding cavity (33). Interestingly, LSD1 inhibition by capsaicin decreases the proliferation, migration and invasion of a gastric cancer cell line (BGC-823) (33), suggesting that this vanilloid compound could have protective roles against this type of cancer. Additionally, some metabolites produced during microbial intestinal transformation of capsaicin and dihydrocapsaicin also display inhibitory effects toward LSD1 (35, 36).

Capsaicin is also linked to the regulation of enzymes that modify DNA methylation. For instance, there are clues that capsaicin may inhibit DNA methyltransferase 1 (DNMT1) (37). Molecular docking simulations showed capsaicin interactions with DNMT1 through hydrogen bonding with G35, Q40, T467 and Q572 residues and establish hydrophobic interactions with L71, W465, Y564 and A568. DNMT1 produces hypermethylation of the genes’ promoter regions coding for the cell adhesion molecule 1 (CADM1) and suppressor of cytokine signaling (SOCS1), which are suppressors of malignant tumor development (38, 39). The HeLa cells (an adenocarcinoma cervical cancer cell line) display DNMT1 overactivity, resulting in the hypermethylation of these genes and downregulation of their expression. Interestingly, HeLa cells exposed to 20 μM capsaicin for a long time showed reversed hypermethylation of CADM1 and SOCS1, restoring the expression of these tumor suppressors. Moreover, high capsaicin treatment produces a decrease in the HeLa cell viability (37). These results suggest that capsaicin could be an anti-methylation agent with the potential for use in the co-treatment of cervical cancer.

The capsaicin’s potential role as an anticancer compound has also been explored in human small-cell lung cancer (40). The long-term treatment of these cells with high capsaicin concentration induces apoptosis through calpain activation; interestingly, this effect dependents on the TRPV6 expression, an abundant receptor expressed in several types of cancer. Similarly, capsaicin produces apoptosis in gastric cancer cells in a TRPV6-dependent manner (41). Since this channel is not directly activated by capsaicin (42, 43), the molecular mechanism of this effect is still unresolved.

The above information briefly exemplifies the capsaicin effects on some cancer cell lines where this compound produces its effects through direct interaction with specific molecular targets. Besides the large number of reports about the antitumoral capsaicin effects, it has also been reported some contrary effects, for example, long-term exposure to low and high capsaicin concentration on some colon and bladder cancer cells (44, 45), positively regulate the epithelial-mesenchymal transition, enhancing their metastatic features, highlighting that we carefully must analyze the benefits and risks of use of capsaicin as a coadjutant in anticancer treatments (30). Therefore, the potential anticancer effects of capsaicin require evaluation for each type of tumor and an appropriate assessment of the optimal concentration and exposure times to obtain greater specificity in the putative anticancer response.

### 3.2 Anti-inflammatory effects of capsaicin: its interaction with inflammatory mediators

Initial studies demonstrated that capsaicin (10 to 50 μM) inhibits the release of prostaglandin E2 from LPS-stimulated peritoneal macrophages. This action is through inhibiting the Cyclooxygenase-2 (COX-2) activity without modifying its expression (46).

Recently, it has shown that capsaicin binds to the COX-2 (47), Pyruvate kinase isoenzyme type M2 (PKM2) and Lactate Dehydrogenase A (LDHA) (48). The report showed that capsaicin inhibited lipopolysaccharide-mediated TNF-𝛼, IL-1𝛽, IL-6 and iNOS expression in the RAW264.7 macrophage cell line, suggesting that capsaicin has anti-inflammatory activity in a TRPV1-independent way since this cell line does not express TRPV1 (48). Interestingly, in a sepsis mouse model treated with capsaicin improved the rate of survival, and upon further exploration with a chemical proteomics approach was discovered that capsaicin exerts its effects through direct interaction with PKM2, LDHA and COX-2 (48). The interaction of capsaicin with these proteins requires key cysteines (C424 of PKM2 and C84 of LDHA), which was confirmed through site-directed mutagenesis studies and molecular modeling (48).

Furthermore, through enzymatic kinetic studies it has also been reported that capsaicin binds to and inhibits human secretory Phospholipase A2 (sPLA2), a key enzyme involved in the hydrolysis of phospholipids to release proinflammatory mediators (47). Molecular modeling showed that capsaicin interaction with sPLA2 is carried out by hydrogen bonds formed between the NH and keto groups of capsaicin with the enzyme residues H47 and G29, respectively, Figure 1D lower. Additionally, the evaluation of the catalytic activity of PLA2 in presence of its substrate and capsaicin showed that the V_max_ was not modified, however, the K_m_ of the enzyme increased in the presence of capsaicin (from 0.14 to 0.25 mM), suggesting that capsaicin competes with the substrate for the binding site in PLA2. Thus, the interaction between capsaicin and PLA2 may prevent the production of inflammatory mediators (47).

The above evidence exemplifies capsaicin’s anti-inflammatory actions; however, it is crucial to consider that capsaicin also has pro-inflammatory effects through TRPV1 activation, this releases Substance P and Calcitonin Gene-Related Peptide, which feeds back the inflammatory response (neurogenic inflammation) (49, 50). Moreover, it has been reported that capsaicin activates human dendritic cells through its classical receptor, TRPV1, having a pivotal role in the immune response (51). These are essential clues to consider when capsaicin is proposed as an anti-inflammatory compound since the immediate effect will always be through TRPV1 activation due to its high affinity, followed by actions on its non-classical targets. Interestingly, the capsaicin long-term exposition of TRPV1 produces its desensitization (52); thus, prolonged use of capsaicin could lead to the loss of its pro-inflammatory effects, with the prevalence of anti-inflammatory effects through non-classical targets.

### 3.3 Capsaicin as regulator of anionic channels and transporters: its putative roles on airway

The effects of capsaicin on the airways have also been explored. It was described that capsaicin potentiates the activity of cystic fibrosis transmembrane conductance regulator (CFTR), an anionic channel regulated by cAMP (53). Electrophysiological experiments carried out in CFTR-expressing cells without TRPV1 expression (NIH-3T3 or CHO cells) showed that capsaicin enhanced cAMP-stimulated CFTR currents. The capsaicin concentration to produce the half-maximal potentiation was calculated as ~48 μM. Interestingly, single-channel recording showed that capsaicin increases the open probability (Po) of CFTR produced by forskolin (53). Furthermore, experiments carried out with genistein, a potentiator of CFTR activity, and high concentration of capsaicin decrease the genistein potentiation of cAMP-stimulated CFTR currents; otherwise, when capsaicin was applied together with low genistein concentration, the CFTR currents were potentiated, suggesting that both compounds act through a common binding site, probably intracellularly located (53).

Capsaicin potentiator effect on CFTR activity has also been reported in the human airway epithelial Calu-3 cells (54). Experiments to measure the apical Cl-currents (aI_Cl_) showed that capsaicin increases the forskolin-induced aI_Cl_, effect reduced by the presence of CFTR inhibitors. Interestingly, capsaicin has opposite effects over several HCO_3_^−^/CI^-^uptake transporters located on the basolateral membrane, indicating that capsaicin strongly impacts the physiology of the airways, and it could be considered for the treatment of CFTR-related inflammatory diseases.

### 3.4 Capsaicin and unexpected effects through TRPV4: its gastrointestinal effects

Recently, the beneficial effects of capsaicin in gastrointestinal physiology have also been reported, (55). The reports show that capsaicin modifies the short-circuit current (I_sc_), which is related to intestinal chloride secretion. Studies achieved in intestinal samples isolated from WT mice showed that I_sc_ stimulated by carbachol and caffeine were attenuated by capsaicin (30 μM) (55). The capsaicin effect was preserved in intestinal samples from TRPV1 knockout mice; however, this effect was lost in the intestine from TRPV4 knockout mice, suggesting that capsaicin attenuates intestinal I_sc_ in a TRPV4-dependent manner. Capsaicin’s inhibitory effects on TRPV4 activity were evaluated in intestinal epithelial cells (IEC-6) and fluorometric assays revealed that capsaicin inhibits TRPV4 activation through its specific agonists (55), indicating that capsaicin inhibits the intestinal CI^-^secretion affecting the TRPV4 function. Interestingly, the jejunal Na^+^ absorption is improved by capsaicin through its inhibitory actions over TRPV4, since reduced Na^+^ absorption is related to diarrhea, capsaicin could act as an antidiarrheal agent (55).

Gastrointestinal function is closer to the vascular system, ensuring adequate blood supply to absorb nutrients and distribute oxygen. Alteration in the vasculature is associated with some inflammatory conditions such as intestinal bowel syndrome (IBS). The endothelial cells play a pivotal role in ensuring vasorelaxation, where endothelium-dependent hyperpolarization (EDH) regulates the resistance vessel and the mesenteric circulation (56). TRPV4 expression is abundant in endothelial cells, and its activation is associated with increased intracellular Ca^2+^ and the concomitant activation of calcium-activated K^+^ channels producing EDH. Peculiarly, capsaicin induces endothelial hyperpolarization through TRPV4 activation, stimulating vasorelaxation. This effect improves blood supply in WT colitis-induce mice but have no effect on the TRPV4 knockout mice (57). These observations allow the use of capsaicin as a natural compound in combination with anti-colitis treatments to improve the health of patients with this illness.

### 3.5 Antiviral capsaicin actions through unclassical partners

Atypical capsaicin roles comprise antiviral effects. Studies performed in A549 cells infected with VSV, H1N1, or ECMV (vesicular stomatitis-, human influenza-, and encephalomyocarditis-virus, respectively) and treated with high capsaicin concentration exhibited marked inhibition of viral replication (58). *In vivo* experiments were also conducted using the VSV intraperitoneal injection in control and capsaicin pretreated mice. The results showed the attenuation of VSV replication in the liver, lung, and spleen of capsaicin-treated mice; capsaicin also reduced the expression of some inflammatory mediators such as TNFα, IlIB, and Il6. Similar experiments were carried out with capsaicin and AMG517, a selective TRPV1 antagonists, which failed to prevent the antiviral capsaicin effect, discarding the TRPV1 involvement.

The mechanism underlines that capsaicin’s antiviral action implicates STAT3, a transcriptional repressor of antiviral immunity. Interestingly, capsaicin induces STAT3 to lysosomal degradation. Apparently, capsaicin interacts in the SH2 domain of STAT3 forming hydrogen bonds with S636 and S623 residues of STAT3 (58). Furthermore, these data agree with a report that demonstrated that capsaicin negatively regulates STAT3 on primary cells of lymphoma and induces the exposition of Damage Associated Molecular Patterns (DAMPs) inducing immunogenic cell death (59); these results demonstrate the effects of capsaicin targeting STAT3 lysosomal degradation enhancing immunity response.

Similar antiviral capsaicin effects have been reported for the porcine enteric coronaviruses. Experiments using IPEC-J2 cells (intestinal porcine enterocytes) demonstrated that the replication of porcine virus required an increase of intracellular Ca^2+^ (60). These cells endogenously express the TRPV4 channel and capsaicin inhibits the calcium entry through this channel and consequently suppress the replication viral (60).

### 3.6 Capsaicin and other unexpected interactions

It has also been reported that capsaicin interacts with collagen (61). The reports found that increasing concentrations of capsaicin reduced the fibril formation (61). Surprisingly, once these collagen fibrils are assembled, capsaicin protects them from enzymatic degradation. Molecular modeling was carried out to infer the interaction of capsaicin with the triple helical conformation of collagen, suggesting that the phenol ring of capsaicin could potentially bind to the “GFOGER,” “EKG,” and “GLO” sequences in the collagen fibril (61).

Peculiarly, capsaicin also has the potential to bind directly to DNA. Using calf thymus DNA (ctDNA) and diverse biophysical tools demonstrated that H-bond and Van der Waals forces are the main interactions between capsaicin and ctDNA. Molecular docking showed that capsaicin bound to ctDNA in the minor groove, placing it in a GC-rich region. Capsaicin-DNA interactions are stabilized by two hydrogen bonds, each with guanine, and have the potential to form hydrophobic interactions with adenine rings (62). The biological effects of capsaicin-DNA interactions remain unresolved, opening a field to research the implications of this interaction and its possible impact on the function of DNA.

## 4 Discussion

Capsaicin is a compound that activates TRPV1, and this function has been intensively studied. Interestingly, there is a large body of evidence showing that capsaicin also impacts cellular functions through different molecular targets, Figure 1C, Table 1. There is clear evidence that upon insertion into cell membranes, capsaicin’s ability to alter the fluidity and stiffness of lipid membranes can significantly modulate ion channels, particularly in cardiac tissue, where capsaicin-induced changes affect cardiac excitability.

**Table 1:** Capsaicin and its unclassical molecular targets: the reported mechanism of action and the biological significance.

Molecular target	Molecular mechanisms of action	Physiological effect	Reference
ASIC1, ASIC3 and P2X2	Capsaicin insertion into plasma membrane	Capsaicin increases the peak current amplitude	Schmidt et al. (18)
Gramicidin A channels (gA)	Capsaicin insertion into plasma membrane	Capsaicin (10–100 μM) increases the activity of the channel Higher concentrations (250 μM) decrease the gA currents	Lundbæk et al. (13) Schmidt et al. (18)
Nav1.5, and inward (Na^+^ and Ca^2+^)/ outward (K^+^) currents	Capsaicin insertion into plasma membrane	Capsaicin inhibits these channels and changes the cardiac excitability	Cowan et al. (20) Isaev et al. (21)
Human Carbonic anhydrase IX (hCA IX) and XII (hCA XII)	Capsaicin interaction in the catalytic binding site establishes electrostatic interactions and H-bound with T200 and T198	Capsaicin inhibits hCAIX/XII, decreasing cell migration and downregulating MMP9 in tumor cells	Gualtieri et al. (23)
Heat shock proteins 90 and 70 (Hsp70 and Hsp90)	Capsaicin binds to the ATP site	Capsaicin reduces the ATPase activity in Hsp90 and down-regulates Hsp70 protein in cancer cells, potential use of capsaicin as anti-tumor agent	Patwardhan et al. (32)
TRPV6	Unknown	Capsaicin induces apoptosis in gastrointestinal-and human small cell lung-cancer cells	Chow et al. (41) Lau et al. (40)
tNOX	Direct capsaicin interaction in an unidentified site	Capsaicin induces tNOX degradation producing apoptosis	Chue et al. (27) Islam et al. (28, 29)
Lysine specific demethylase 1A (KDM1A/LSD1)	Capsaicin binds to the FAD cavity of LSD1 and overlaps in the catalytic cavity in a reversible manner	Capsaicin inhibits LSD1 activity in gastric cancer cells, decreasing proliferation and migration	Jia et al. (33)
DNA-methyltransferase 1 (DNMT1)	Interactions involving Gln40, Leu71, Tyr564	Capsaicin generates an inhibitory effect on *DNMT1*. Consequently, it reverses the hypermethylation in *CADM1* and *SCOS1*, reducing cell proliferation and tumorigenesis in cervical cancer	Sharan et al. (37)
Cyclooxygenase-2 (COX-2) Pyruvate kinase isoenzyme type M2 (PKM2) L-lactate dehydrogenase A (LDHA)	Capsaicin modifies covalently cysteine residues of these enzymes	Capsaicin has anti-inflammatory effect in LPS-induced macrophages or sepsis-related inflammation *in vivo*	Kim et al. (46) Zhang et al. (48)
Phospholipase A2 (PLA2)	Capsaicin forms hydrogens bonds with H47 and G29 in the active site of PLA2	Anti-inflammatory activity	Aiswarya, et al. (47)
Cystic fibrosis transmembrane conductance regulator (CFTR)	Capsaicin possibly binds on the cytoplasmic side; it may be by a dimerization in the NBD	Positive regulator of CFTR. Treatment against disorders of cystic fibrosis patients	Ai et al. (53)
TRPV4	Unknown	Capsaicin inhibits the intestinal Cl-secretion affecting the TRPV4 function. Capsaicin also induces endothelial hyperpolarization through TRPV4 activation, stimulating vasorelaxation. Potential treatment against diarrhea and ulcerative colitis	Wan et al. (55) Zhang et al. (48)
STAT3	Interacts directly with STAT3 in the SH2 domain.	Inhibition of < STAT3 activation Restriction of viral replication by enhancing the type I IFN response	Zhang et al. (48)
Collagen fibers	Bind to collagen fibers via the aromatic moiety and amide region of the capsaicin in the triple-helical collagen (“GFOGER,” “EKG” and “GLO”)	Suppression of the assembly of collagen fibrils assembly and protection of collagen fibrils from enzymatic degradation	Perumal et al. (61)
Minor groove of the DNA strand	Spontaneously interacts through non-intercalative groove binding mode involving hydrogen bond and Van der Waal interactions.	Unknown	Qais et al. (62)

Beyond its role in sensory perception, capsaicin has shown promising anti-cancer properties via interactions with several cancer-related mediators, positioning it as a potential complementary agent in cancer treatment. Capsaicin also has promising potential as an anti-inflammatory agent due to its ability to inhibit key enzymes involved in inflammation, modulating these pathways, capsaicin may have therapeutic implications in inflammatory-related conditions such as sepsis and chronic inflammatory diseases.

In summary, capsaicin is far more than just a spicy compound; it is a bioactive molecule with diverse and complex effects on mammalian physiology, and thus, it is essential to study all the possible functions outside its interactions with TRPV1 in more detail. Its ability to regulate membrane dynamics, modulate enzymatic activity, and influence ion channel’s function, positions it as a potential therapeutic agent in fields like cardiology, oncology, immunology, and infectious disease treatment, Table 1. While further research is still needed to harness its benefits fully, capsaicin’s multifaceted actions make it an intriguing candidate for future biomedical applications.

### 4.1 Future perspectives

Capsaicin, used as a natural co-adjuvant to relieve some pathological conditions, should considered the proper use of this compound since high concentrations can accumulate in the plasma membrane and produce collateral harmful consequences, such as cardiovascular alterations.

Curiously, some of the unclassical capsaicin interactions are beyond transmembrane localization, suggesting that capsaicin would have to be able to achieve the nucleoplasm to interact with LSD1 and DNMT1 or be available in the cytoplasm to exert its actions over some enzymes as Hsp90. It will be possible through the capsaicin accumulation in the plasma membrane and its internalization through endocytic vesicles, where the capsaicin would be inserted in the “tail-up” and “head-down” configuration, leaving accessible the head and neck domains to establish H-bond with the amino acids of its intracellular molecular targets, which is a typical interaction between this capsaicinoid and the proteins before mentioned.

Another interesting perspective is detailing the molecular mechanism of capsaicin action over the function of other TRPV channels, impacting gastrointestinal and cancer cell physiology. The capsaicin insertion to membranes could be the mechanism to regulate the function of TRPV4 and TRPV6 since these channels also play a role as mechanosensors (63, 64), thus any modification on the membrane fluidity could affect their function; it will be interesting to assay the function of these channels inserted in artificial membranes applying low and high concentrations of capsaicin to determinate how the capsaicin insertion into membranes affects the function of these channels.

Finally, the main future perspective of using capsaicin is having an efficient delivery system, and the nano micelles could be a great option. Thus, further research is required to find the optimal use of capsaicin beyond an analgesic alternative.
